# Oxidation Impacts the Intracellular Signaling Machinery in Hematological Disorders

**DOI:** 10.3390/antiox9040353

**Published:** 2020-04-24

**Authors:** Elena Tibaldi, Enrica Federti, Alessandro Matte, Iana Iatcenko, Anand B. Wilson, Veronica Riccardi, Mario Angelo Pagano, Lucia De Franceschi

**Affiliations:** 1 Department of Molecular Medicine, University of Padua, 35131 Padua, Italy; elena.tibaldi@unipd.it (E.T.); mario.pagano@unipd.it (M.A.P.); 2 Department of Medicine, University of Verona and AOUI Verona, 37134 Verona, Italy; federti.enrica@gmail.com (E.F.); alessandro.matte@gmail.com (A.M.); ianaiatcenko@gmail.com (I.I.); anand.wilson16@gmail.com (A.B.W.); veronica.riccardi@studenti.univr.it (V.R.)

**Keywords:** Src family kinases, protein phosphatase, Lyn, red cells, sickle cell disease, chronic lymphocytic leukemia, erythropoiesis, thalassemia, G6PD deficiency

## Abstract

The dynamic coordination between kinases and phosphatases is crucial for cell homeostasis, in response to different stresses. The functional connection between oxidation and the intracellular signaling machinery still remains to be investigated. In the last decade, several studies have highlighted the role of reactive oxygen species (ROS) as modulators directly targeting kinases, phosphatases, and downstream modulators, or indirectly acting on cysteine residues on kinases/phosphatases resulting in protein conformational changes with modulation of intracellular signaling pathway(s). Translational studies have revealed the important link between oxidation and signal transduction pathways in hematological disorders. The intricate nature of intracellular signal transduction mechanisms, based on the generation of complex networks of different types of signaling proteins, revealed the novel and important role of phosphatases together with kinases in disease mechanisms. Thus, therapeutic approaches to abnormal signal transduction pathways should consider either inhibition of overactivated/accumulated kinases or homeostatic signaling resetting through the activation of phosphatases. This review discusses the progress in the knowledge of the interplay between oxidation and cell signaling, involving phosphatase/kinase systems in models of globally distributed hematological disorders.

## 1. Introduction

In the last decade, growing evidence has highlighted the importance of reactive oxygen species (ROS) interfacing intracellular signaling pathways to ensure cell survival [1,2]. This requires a balance between oxidation and cytoprotective systems to maintain cell homeostasis and to limit the severity of oxidative stress. In addition, ROS might promote the transient oxidation of cysteine groups on proteins involved in signaling networks, contributing to protein conformational changes and affecting cell signaling. This results in either a block of protein function such as in protein tyrosine phosphatase (PTP) or protein phosphatase 1 and 2 (PP1, PP2A), or activation of protein kinases such as Src family kinases (SFKs) [3,4,5]. Furthermore, oxidation might directly induce/modulate kinase activity such as in Src family kinases (SFKs) or Akt serine-threonine kinase [6,7,8,9,10,11]. Translational studies have revealed the important link between oxidation and signal transduction pathways in both oncologic and benign hematological disorders [12,13,14,15,16,17,18,19,20,21]. In this review, we focus on models of globally distributed hematological diseases for which the link between intracellular signaling and oxidation has been recently reported. Among them, we plan to discuss (i) chronic lymphocytic leukemia (CLL), as a model of onco-hematological disease; (ii) β-thalassemia, as a model of pathologic erythropoiesis; and (iii) sickle cell disease (SCD) and G6PD deficiency as models of inherited red cell disorders characterized by severe membrane oxidative damage. 

CLL is a worldwide distributed leukemia with a highly heterogeneous clinical presentation. In CLL, CD5^+^/CD19^+^/CD23^+^ B lymphocytes proliferate in lymphoid tissues and bone marrow (BM), accumulating as mature quiescent cells [22,23,24]. Studies in CLL have shown the crucial role of the bone marrow microenvironment and secondary lymphoid organs, where T cells, monocyte-derived nurse-like cells, and mesenchymal stromal cells sustain proliferation and pro-survival mechanisms. These are triggered by B cell receptor (BCR) signaling together with the activation of NF-kB [25,26,27]. Intriguingly, ROS are abundantly produced in CLL cells mainly by mitochondria [28,29]. However, the elevated oxidative potential is counteracted by a marked ROS-buffering capacity provided by both antioxidant enzymes and the support of the microenvironment in lymphoid tissues [28,29].

BM microenvironment is also important to support normal and pathologic erythropoiesis [30,31]. β-thalassemia is a globally distributed, hereditary erythroid disorder, caused by the absence or decreased production of the β-globin chain. This results in a chronic hemolytic anemia linked to reduced red cell survival and ineffective erythropoiesis [32,33]. Previous studies have shown that chronic and severe oxidative stress plays a crucial role in the pathogenesis of anemia of β-thalassemia [32,33]. This is mainly related to the erythroid accumulation of free α-globin chains and free heme, associated with a perturbation of iron homeostasis [34,35]. SCD is one of the most common monogenic red cell disorders. SCD is characterized by the synthesis of the pathological hemoglobin S (HbS). HbS shows unique biochemical properties, polymerizing when deoxygenated. This results in abnormal red cell membrane ion permeability with generation of dense, dehydrated erythrocytes, which accelerates HbS polymerization [36,37]. This also sustains severe membrane oxidative damage, which contributes to reduced sickle red cell survival into the peripheral circulation [36,37]. G6PD deficiency is another worldwide distributed hereditary red cell disease [38,39]. The inability of G6PD deficient erythrocytes to be protected against increased oxidation has been mainly linked to their incapacity to remove peroxides through the glutathione peroxidase/reductase system [38,39]. This review discusses the progress in the knowledge of the interplay between oxidation and cell signaling, involving phosphatase/kinase systems in models of hematologic oncologic and benign worldwide distributed disorders.

## 2. Abnormal Intracellular Signaling and Oxidative Stress Characterizes CLL 

### 2.1. Oxidation Affects the Dynamic Coordination between Kinases and Phosphatases in Cancer Cells

Tumor cells and immune cells that infiltrate tumors or partake in the tumor microenvironment have been described to generate large amounts of ROS, which impact on numerous cancer processes such as proliferation, survival, or cell apoptosis [40,41]. Whether generated as by-products by mitochondria or by plasma-membrane oxidoreductases such as NADPH oxidases, ROS can cause damage directly to deoxyribonucleic acid (DNA), proteins, and lipids, as well as alter the activity of numerous key signaling molecules including protein tyrosine phosphatases (PTPs) and protein tyrosine kinases such as SFKs [42,43,44]. Among SFKs, Src and Lyn play a central role in the early steps of the signaling cascade by targeting downstream substrates. SFKs have been described to be overactive in different cancers, supporting cancer cell survival by antiapoptotic mechanisms. SFKs are also subject to several mechanisms of regulation, such as intracellular interactions and post-translational modifications [45]. Indeed, SFKs are susceptible to redox regulation due to highly conserved redox-sensitive cysteines, which is demonstrated by the increased catalytic activity under intracellular oxidative conditions, including cancer [46,47]. This occurs not only in the catalytic domain, but also within Src homology 2 (SH2) domains domains, which mediate the interaction of SFKs with phosphor-Tyr-containing substrates, receptors, or adapters [48]. Notably, SFKs contribute to increasing ROS levels by phosphorylating, and thereby enhancing the activity of, NADPH oxidases (NOXs), reinforcing the effects of an oxidative environment [49]. Whereas oxidation-mediated overactivation of SFKs can be regarded as one of the causes of tonic phosphorylation-dependent signaling in cancer cells, the inhibition of PTPs has been considered even more important in cancer signaling [48]. PTPs harbor a conserved HC(X)_5_R catalytic motif, with the catalytic cysteine having a low pKa, which makes them sensitive to inactivation by ROS [42]. In cancer cells, this cysteine is likely to become oxidized, resulting in phosphatase activity inhibition and unbalanced kinase activity, which further sustain pro-cell survival cancer signals.

### 2.2. Imbalance between Kinases and Phosphatases Activities: The Key Mechanism Underlying CLL Development

CLL is characterized by mature clonal B lymphocytes arrested in the G0/G1 phase of the cell cycle in the peripheral blood and a smaller cell population that actively proliferates in the secondary lymphoid organs [50,51]. Importantly, many of the functional characteristics of CLL cells have been shown to depend on BCR signaling, which can be broadly defined as antigen-independent (tonic) and antigen-mediated [50]. Such signals are generated by a “signalosome” located immediately downstream of the BCR involving constitutively activated kinases, such as Lyn, Syk, Btk, PI3K, and Akt, as well as adapter proteins, e.g., B-cell linker protein (BLNK) and growth factor receptor-bound protein 2 (GRB2) [50,52,53]. This multiprotein complex in turn propagates the oncogenic signals through the activation of different downstream signaling cascades, including mitogen-activated protein (MAP) kinase and the canonical NF-kB signaling pathways, eventually leading to the upregulation of the antiapoptotic proteins MCL1, BCL-XL and XIAP [54,55]. This scenario is further complicated by the fact that Lyn occurs in two constitutively active forms, one as a component of the above-mentioned signalosome and the other aberrantly located in the cytosol as part of a soluble complex, which contributes to massive tyrosine phosphorylation, and hence the functionality of its downstream substrates [12,56]. Importantly, few of the kinases mentioned above have been also regarded as potential therapeutic targets for CLL. This resulted in development of two drugs that selectively inhibit BTK and PI3K, ibrutinib and idelalisib, respectively, which are now administered as second-line regimens in CLL [12,57]. Up to now, accumulating evidence has increasingly demonstrated that the anomalous phosphorylation-mediated signal transduction in CLL also depends on the inability of the functional counterparts of kinases, namely phosphatases, to exert a negative control over constitutively activated signaling pathways. In normal B cells, protein phosphatases (e.g., Src homology 2 domain-containing phosphatase 1, SHP-1) and lipid phosphatases (e.g., phosphatase and tensin homolog, PTEN) are recruited in proximity to the BCR and contribute to a feedback inhibitory circuit, dephosphorylating proximal BCR signaling intermediates such as kinases and relevant targets, thereby negatively regulating signaling [58,59,60]. Instead, in CLL there are several phosphatases that are dysregulated due to a decreased expression, including PTEN, protein tyrosine phosphatase receptor-type O truncated (PTPROt), PH domain and leucine rich repeat protein phosphatases (PHLPP1) and Src homology region 2 domain containing inositol polyphosphate 5-phosphatase 1 (SHIP1), or due to overexpression, which is the case for tyrosine phosphatase non-receptor type 22 (PTPN22), which has been shown to support antiapoptotic signals by positively affecting B-cell receptor-dependent signaling pathways [56,57,58,59,60]. Other phosphatases, such as serine/threonine protein phosphatase 2A (PP2A) and SHP-1 are expressed to an extent similar to that observed in normal B cells, their activity being, however, downregulated by several mechanisms, which are mainly mediated by the abnormally active Lyn kinase [61,62]. PP2A inhibition has been shown to be due to the interaction with the endogenous inhibitor SET, which is strengthened by the phosphorylation of its catalytic subunit, and its phosphatase activity can be restored by using compounds that remove SET itself. On the other hand, SHP-1 exists in CLL cells as two distinct pools, one of which is bound to the Tyr-phosphorylated cytoplasmic tail of the transmembrane CLL marker CD5, most likely supporting pro-survival pathways, and the other that is located in the cytoplasm in an inactive form that can be reactivated by small molecules that disrupt the inhibitory intramolecular interactions. Importantly, the restoration of the activity of these two phosphatases has been reported to evoke CLL cell apoptosis by disrupting the phosphorylation-dependent pro-survival and antiapoptotic signaling network [13,62]. In this regard, we recently showed that compounds restoring the activity of PP2A and SHP-1, which is largely impaired in varied types of cancer, constrained kinase-orchestrated survival signaling pathways, thereby inducing apoptosis in CLL cells; notably, the use of such activators in combination with kinase inhibitors resulted in a dramatic synergistic effect [12,63], which may open a new avenue in the development of new therapeutic strategies.

#### 2.2.1. CLL Cells Exhibit High ROS-Buffering Capacity to Preserve Survival 

**Figure 1:**
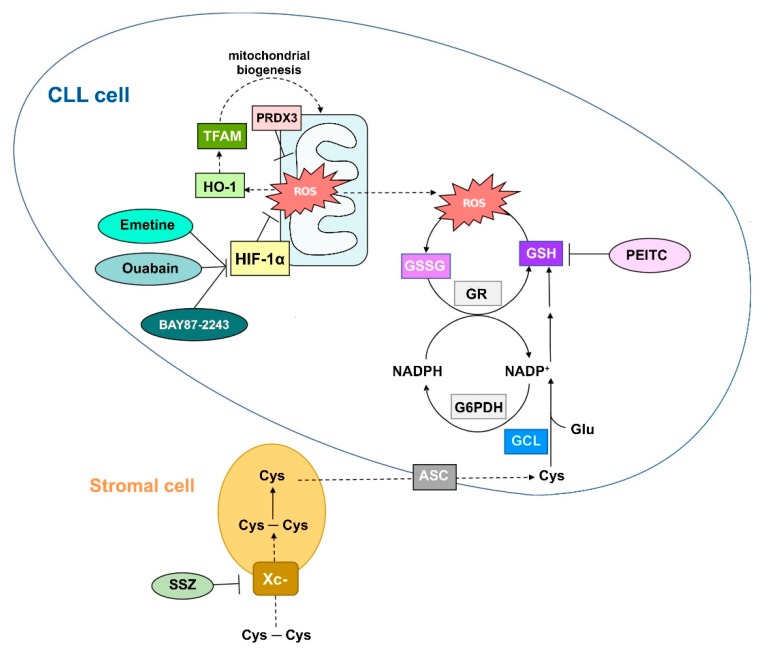
Schematic diagram showing key antioxidant systems in chronic lymphocytic leukemia (CLL) cells and small molecules capable of abolishing the redox balance, resulting in reduced CLL cell vitality. ASC, alanine-serine-cysteine transporter; Cys, cysteine; Cys-Cys, cystine-cysteine; G6PDH, glucose-6-phosphate dehydrogenase; GCL, γ-glutamyl cysteine ligase; GSH, glutathione; GSR, glutathione reductase; GSSG, glutathione disulfide; Glu, glutamate; HIF-1α, hypoxia-inducible factor-1α; HO-1, heme oxigenase-1; NADP+/NADPH, nicotinamide adenine dinucleotide phosphate; PEITC, β-phenylethyl isothiocyanate; PRDX3, peroxiredoxin 3; SSZ, sulfasalazine; Xc-, cystine transporter.

As in other cancer cells, CLL cells must adapt to their increased metabolic demands by boosting their mitochondrial activity and enhancing mitochondrial biogenesis, resulting in oxidative stress through the sustained generation of ROS [28,29,63]. Oxidative stress in CLL cells has thus far not been shown to affect either kinase or phosphatase activities. This distinguishes CLL cells from normal B cells, where BCR engagement, in addition to initiating signaling by activating SFKs and Syk, also results in the transient inhibition of SHP-1 through oxidation of the catalytic cysteine by H_2_O_2_. H_2_O_2_ is generated in the cell exterior by the coupled action of NADPH oxidase 2 (NOX2) and an extracellular form of superoxide dismutase, which generates superoxide anions and, in turn, O_2_^•−^. This latter ROS is produced by NADPH oxidase 2 (NOX2) and released, where it is subsequently taken up by the activated B cell through a transmembrane channel, identified as aquaporin 8 [64]. The role of NOX2 and the downstream generation of ROS as secondary messengers has been shown to be essential for amplifying kinase-mediated signals [65,66], and is especially requireds for B cell proliferation [67]. Interestingly in this regard, BCR-dependent constitutively active signaling in CLL is scarcely related to ROS production by the plasma membrane-bound NOX2, the condition of which may also depend on the lower expression of the catalytic subunit of the oxidase itself in these leukemia cells [63]. In response to oxidation, CLL cells activate a complex antioxidant machinery that is strongly supported by the tissue stromal micro-environment, as evidenced by the prolonged survival in vivo compared to the spontaneous apoptosis in vitro of CLL cells themselves [68,69]. A crucial pathway being the Keap1-Nrf2-ARE pathway, which upregulates heme-oxygenase–1 (HO-1) and catalase, among others. Whereas catalase directly detoxifies H_2_O_2_ to water and oxygen, HO-1 acts as a positive regulator of TFAM, a mitochondrial transcription factor, in turn stimulating de novo mitochondrial biogenesis, which is aimed at compensating for the damage induced by ROS and the consequent decrease in energy production [63,64,65,66,67,68]. Moreover, the ROS-scavenging capacity of CLL cells is also to be ascribed to thiols, which are significantly more abundant than in normal B cells [68]. Glutathione (GSH) substantially contributes to the content of thiols in CLL cells, which is supported by increased levels of enzymes that are directly involved in GSH biosynthesis and recycling, namely γ-glutamyl cysteine ligase (GCL), the rate limiting enzyme, and glucose-6-phosphate dehydrogenase (G6PDH), the key enzyme in the pentose phosphate pathway which provides NADPH, the co-factor of GSH reductase [63]. Notably, a critical role in de novo GSH biosynthesis is also played by the stromal micro-environment, which supplies cysteine, the limiting factor in this process, to CLL cells. The constant need for this amino acid cannot be met by the limited amounts in the extracellular environment, where cysteine itself is unstable and swiftly oxidized to cystine. Moreover, CLL cells scarcely express xCT, the catalytic subunit of the cystine transporter Xc-, which is instead abundant in stromal cells. These latter cells can convert cystine to cysteine, which is released and immediately taken up by CLL cells through the alanine-serine-cysteine (ASC) transporter to synthesize GSH and support cell survival (Figure 1) [67].

Furthermore, direct contact with stromal cells induces the expression of peroxiredoxin 3 (PRDX3), which is mostly located in the mitochondria of CLL cells, where it regulates the abundance of H_2_O_2_. 

Not less important in countering oxidative stress is Hypoxia-Inducible Factor α (HIF-1α), a transcription factor that regulates its target genes both in the nucleus and in the mitochondria [70]. Upon contact with stromal cells, HIF-1α is upregulated in CLL cells by virtue of the miRNA-mediated downregulation of von-Hippel–Lindau (VHL) ubiquitin ligase, which instead targets HIF-1α for degradation under normoxia [71]. A HIF-1α-mediated mechanism countering oxidative stress is known to rely on the decrease of mitochondrial complex I activity by both inducing transcription of miRNAs that repress the expression of the iron–sulfur cluster assembly protein ISCU1/2 [72] and fostering expression of NDUFA4L2, thereby reducing oxygen consumption and ultimately decreasing the production of ROS (Figure 1) [73].

#### 2.2.2. Potentiation of Antioxidant Defense Systems and CLL 

In light of the heterogeneity and the fatal progression of CLL as well as the emerging resistance to the front and second-line drugs currently in clinical use, new therapeutic options are needed so as to improve the outcome of CLL patients [74,75,76]. In this regard, oxidative stress and the protective mechanisms that CLL cells enact have recently received considerable attention as novel therapeutic targets. A few small molecules have been tested in vitro and in vivo, proving effective in eliciting apoptosis of CLL cells not only by subverting their antioxidant machinery but also interfering with the protective role of the micro-environment. For instance, the cardiac glycoside ouabain and the ipecac alkaloid emetine perturb intracellular redox homeostasis in the low nanomolar range by repressing HIF-1α, resulting in a dramatic rise of ROS and apoptosis of CLL cells [29]. The HIF-1α selective inhibitor BAY87-2243 has been shown to exert cytotoxic effects on CLL cells as such and to strongly synergize with the BTK inhibitor inbrutinib or fludarabine, offering new prospects in terms of translational impact. Regarding fludarabine, CLL cells frequently exhibit resistance to this purine analogue, which can be overcome by β-phenylethyl isothiocyanate (PEITC), a naturally occurring compound that causes rapid depletion of cellular glutathione, resulting in increased ROS, oxidative stress, and cell death [67]. Overlapping effects take place by using sulfasalazine (SSZ), a drug that can be used in the treatment of inflammatory diseases, which inhibits Xc- in stromal cells, consequently impeding the conversion of cystine into cysteine, which is key to de novo GSH biosynthesis in CLL cells [67]. 

Further research is warranted to broaden our knowledge of the factors involved in ROS generation and detoxification in CLL cells, but the few above-mentioned examples provide evidence of the considerable potential of treatments affecting the redox balance in cancer cells.

## 3. Erythropoiesis: The Interplay between Oxidation and Signal Transduction Pathways

Erythropoiesis is a multistep process with high biocomplexity, ensuring cell differentiation and maturation from committed erythroid progenitors to erythroblasts that then become reticulocytes finally maturing into red cells that enter the peripheral circulation. Although in erythroid commitment the signal transduction cascade defined as the erythropoietin (EPO) pathway is crucial for erythroid maturation, it seems that other kinase(s) beside Jak2 kinase can be involved such as Lyn or Fyn [35,77,78]. Indeed, Lyn^−/−^ and Fyn^−/−^ mice show perturbation of late stage erythropoiesis associated with increased oxidation and cell apoptosis [11,78,79,80,81]. 

Erythroid differentiation is associated with the production of ROS both in response to the EPO cascade and to the presence of free iron required for the biosynthesis of heme [34,82,83,84]. Previous studies have reported that oxidation activates both Akt and Fyn, thus involving ROS in modulation of signaling pathways during erythropoiesis [11,35,85]. Indeed, the importance of controlling oxidation during erythroid maturation is also supported by the abnormalities of hematologic parameters of superoxide dismutase 2 (SOD2) null embryos and by the chronic hemolysis of peroxiredoxin 2 (Prx2) knockout mice associated with ineffective erythropoiesis [83,86,87,88]. 

### 3.1. Redox Interfaces with Cell Signaling in Erythropoiesis 

**Figure 2:**
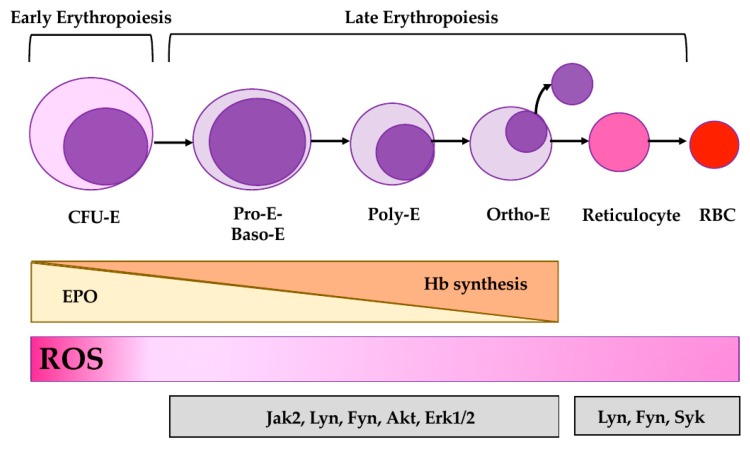
Schematic diagram of erythropoiesis. Early phase of erythropoiesis ends with the cell forming erythroid units (CFU-E), whereas the late phase of erythropoiesis starts with pro-erythroblasts through basophilic, polychromatic, and orthochromatic erythroblasts (E) towards enucleation with generation of reticulocytes, maturating into erythrocytes or red blood cells (RBCs). Erythropoiesis is regulated by erythropoietin (EPO), the signaling of which is associated with reactive oxygen species (ROS) production. The presence of free iron required for the biosynthesis of heme and the production of hemoglobin (Hb) contributes to oxidation in erythropoiesis. Jak2 kinase and Src family kinases (Lyn and Fyn) are involved in the EPO cascade. Redox interfaces these kinases and Akt towards mTOR-dependent autophagy.

In stress erythropoiesis, such as in β-thalassemic syndromes or in phenylhydrazine (PHZ) induced hemolytic anemia, the prolonged/severe oxidation results in abnormal intracellular signaling (Figure 2) [11,35,85,89,90,91]. This is characterized by increased activation of Jak2, associated with the over-activation of Akt and Fyn kinases [11,35,85,89,90,91]. Akt is important in several signaling pathways which are crucial against oxidation or important in cell homeostasis [85,92,93,94,95]. This is mainly driven by the phosphatidylinositol-4, 5- biphosphate 3 kinase (PI3K)/Akt pathway, involved in the regulation of the synthesis of 1, 3-bisphosphoglycerate (BPG) that is part of glycolysis [85,92,93,94,95]. In β-thalassemia, Akt in turn activates the mammalian target of rapamycin (mTOR), blocking autophagic processes with the accumulation of damaged proteins such as the highly pro-oxidant free-alpha globin chains. This blocks erythroid cell maturation, promoting cell apoptosis with ineffective erythropoiesis [11,35,96]. As a proof of concept, rapamycin, an mTOR inhibitor, improves pathologic erythropoiesis in a mouse model of β-thalassemia, whereas rapamycin worsens normal erythropoiesis in wild-type mice similar to what is observed in mouse model genetically lacking mTOR [97]. These results support the importance of the functional link between redox and cell signaling during erythropoiesis [11,35,96]. Fyn is an SFK highly expressed in hematopoietic cells [6,9,11,98,99]. The function of SFKs might be modulated by redox, which increases Tyr416 phosphorylation resulting in the activation of the SFKs [6,9,11,98,99]. In addition, the oxidation of Cys245 and 487 induces a protein conformational change, facilitating the activation of SFKs [6,9,11,98,99]. Recently, Beneduce et al. have shown that Fyn is in a crossroad between EPO induced oxidative stress, required for erythroid maturation and regulation of Nrf2 function, which regulates the expression of antioxidants and cytoprotectors such as HO-1, catalase or peroxiredoxins (Figure 2) [11]. Indeed, Fyn plays a pivotal role in post-induction of Nrf2 activation against oxidation [11,100,101]. In stress erythropoiesis, prolonged activation of Nrf2 is associated with the accumulation of inactive/damaged proteins, contributing to impairment of autophagy. These proteins promote oxidation of erythroid maturating cells, resulting again in a block of cell maturation and ineffective erythropoiesis [11]. In mice genetically lacking Fyn, rapamycin beneficially affects stress erythropoiesis, further reinforcing the importance of the modulation of the redox interface with cell signaling towards autophagy as an adaptive mechanism against severe or prolonged oxidation. Notably, Fyn has been also reported as an additional regulatory kinase for the thrombopoietin-Jak2 pathway during megakaryocytopoiesis, reinforcing the central role of Fyn in hematopoiesis dealing with oxidation and signaling in cell maturation processes [102]. Among the Ser-Thr kinases linked to EPO signaling and affected by oxidation, the extracellular signal-regulated kinase (Erk)-1 and -2 seem to negatively regulate cellular differentiation in the early stage of erythropoiesis [100,101]. In vitro studies on human β-thalassemic erythropoiesis have shown abnormal activation of Erk1/2 kinases, which may possibly target the Bax/Bcl2 system, promoting either cell proliferation or cell apoptosis in the early and late phase of β-thalassemic erythroid maturation process, respectively [100,101]. 

Notably, reticulocytes dismissed from the BM still express SFKs such as Lyn, Fyn, Fgr, or HcK as well as Syk, a Src related kinase [5,6,10,19]. In diseased red cells such as SCD, kinases such as Erk1/2 have been reported to contribute to adhesive events between red cells and inflammatory activated vascular endothelial surface [103,104]. The presence of a signaling machinery in red cells further supports the connection between oxidation and intracellular signaling pathways. These are involved in the modulation of membrane ion transport systems or in membrane protein rearrangement against oxidation to ensure red cell survival and aging (Figure 2). 

### 3.2. Antioxidant System(s) and Stress Erythropoiesis

The control of the detrimental effects of oxidation during erythropoiesis is extremely important to ensure cell differentiation and maturation (Figure 2) [86,105]. Previous studies have shown that prolonged or severe oxidation such as in stress erythropoiesis requires either the potentiation of endogenous antioxidant systems or the treatment with exogenous antioxidants. Recently, we reported the key role of peroxiredoxin-2 (Prx2) in normal and pathologic erythropoiesis [83,86,87,106]. Prx2 might act as an antioxidant and atypical molecular chaperone targeting free heme to control severe oxidation [82,83,106]. Indeed, the beneficial effects of the administration of recombinant PEP1-Prx2 fusion protein on anemia of a mouse model of β-thalassemia further supports the importance of an antioxidant system against chronic oxidation in pathologic erythropoiesis [83,87]. Notably, exogenous antioxidants such as resveratrol or monoHER (7-monohydroxyethylrutoside), a semi-synthetic flavonoid antioxidant, reduce oxidation and improve ineffective erythropoiesis in both mouse models for β-thalassemia and in vitro human β-thalassemic erythroid cells [82,107]. Another possible strategy to limit oxidation in pathologic erythropoiesis is to reduce the free pathologic heme. In β-thalassemic mice, we recently reported that bitopertin, a specific glycine transport inhibitor, ameliorates murine β-thalassemic erythropoiesis with the activation of autophagy and improvement of quality control processes during β-thalassemic erythropoiesis [34]. Taken together these data support the importance of modulation/potentiation of an endogenous antioxidant system as a novel therapeutic strategy against severe/prolonged oxidation in normal and pathologic erythropoiesis. 

## 4. Redox Affects Signaling Pathways Involved in Red Cell Homeostasis 

### 4.1. Oxidation and Signal Transduction Pathways in Red Cell Pathologies

**Figure 3:**
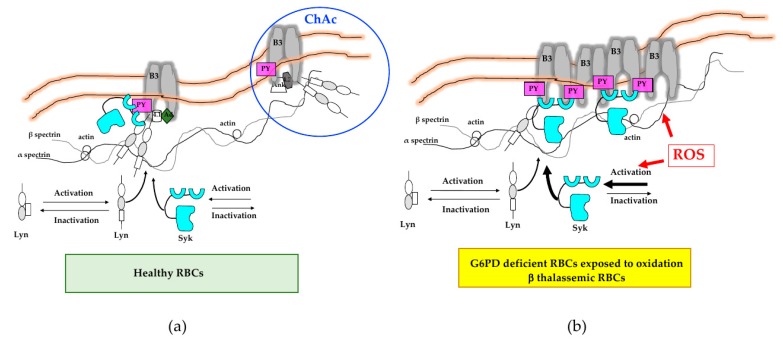
Schematic diagram of the main cell signaling pathway involved in red cell homeostasis. (**a**) Under steady state conditions, Lyn and Syk are inactive. Cellular stress such as physiologic oxidation related to aging or osmotic stress triggers Lyn–Syk sequential activation, resulting in increased tyrosine (Tyr) band 3 (B3) phosphorylation (PY). This modulates B3 interaction with neighboring proteins such as band 4.1, band 4.2, or adducin or ankyrin. These multiprotein complexes bridging the membrane to the cytoskeleton contribute to membrane mechanical instability. Red cells from patients with chorea-acanthocytosis (ChAc) loose the canonical Lyn–Syk signaling pathway targeting B3, resulting in membrane–cytoskeleton network instability. (**b**) Red cells from patients with either G6PD deficiency exposed to oxidation or β-thalassemia are characterized by severe oxidation. This results in membrane damage and abnormal activation of Syk independently from the canonical Syk–Lyn pathway. The damaged, over-Tyr phosphorylated B3 forms clusters, which favor erythroid vesicle release and the fast removal of the damaged red cells by splenic macrophages.

In erythrocytes, the importance of intracellular signals against exogenous/endogenous oxidative stress is supported by evidence in mouse models genetically lacking either phosphatases such as PTPε or kinases such as Lyn or Fyn [5,10,11,108]. The absence of either PTPε or Lyn or Fyn results in mild hemolytic anemia associated with reduced red cell survival or abnormalities in red cell density and volume regulation mechanisms [5,10,11,108]. Studies in human normal and diseased red cells have led to the development of a general model of erythroid cell membrane organization based on the cross-talk between the membrane lipid bilayer, the integral membrane proteins such as band 3, and the peripheral proteins such as alpha/beta spectrins (Figure 3, left panel). The band 3-based bridges formed by multiprotein complexes involving ankyrin and protein 4.1 (junctional complex) anchor the red cell membrane to the skeleton network (Figure 3, left panel) [19,109,110]. 

In erythrocytes, the imbalance of phosphatase–kinase activities might result in abnormal band 3 Tyr-phosphorylation state associated with reduced membrane mechanical stability [14,15,17,19,111,112,113]. In erythrocytes exposed to cellular stresses, such as physiologic oxidation or osmotic stress, Syk and Lyn are sequentially activated, targeting band 3 respectively on Tyr residue 8 and 904. This affects band 3 protein interactions with membrane associated proteins and/or with proteins of the cytoskeleton network [5,10,11,108]. In red cells from patients with chorea-acanthocytosis (ChAc), a rare hereditary neurodegenerative disorder characterized by the presence of circulating acanthocytes, we showed accumulation of active Lyn independently from its canonical signaling cascade through the primary activation of Syk. We then demonstrated that the ChAc-associated alterations in red cell membrane protein organization are generated by the altered linkage of band 3 to the junctional complexes, with production of acanthocytes. This conclusion is also supported by the presence of acanthocytes in mice genetically expressing active Lyn [14,15,80]. 

Studies in G6PD deficient red cells exposed in vitro to diamide, β-thalassemic erythrocytes or sickle erythrocytes undergoing in vitro deoxygenation, have had documented an over-activation of Syk bypassing the canonical cascade and targeting clustered band 3 [17,18,107,114,115,116,117]. This favors the release of erythroid microparticles and the macrophage biting during transit of pathologic red cells in spleen microcirculation (Figure 3, right panel). Indeed, pharmacologic active Syk inhibitors such as imatinib in association with artemisinin have been shown to accelerate the clearance of malaria parasitized red cells by splenic macrophages [17,106,114,116,117]. Up to now, a clinical study on the effects of the combination imatinib-artemisinin treatment in patient with malaria is on-going in Vietnam (NCT#02614404). 

### 4.2. Functional Interplay between Oxidation and Cell Signaling Towards Regulation of Erythrocyte Volume 

The proper function and survival of red cells in peripheral circulation requires the maintenance of optimal cell volume. This is regulated by the activity of various membrane ion transport pathways and channels, the large part of which is regulated by phosphorylation/dephosphorylation events [16,19,118]. The Na-K ATPase pump is characterized by alpha and beta subunits, the function of which depends on ATP concentration and is modulated by protein kinase A (PKA) and C (PKC) activities [118,119]. In erythrocytes from SCD patients, increased activity of PKCs has been reported in reticulocytes but not in dense and older erythrocytes, which are characterized by loss of PKC activity, contributing to the abnormalities of Na-K pump activity observed in SCD erythrocytes [10,118,120,121]. The Na-K-2Cl (NKCC) cotransport is another transport system, modulated by a cyclic phosphorylation/dephosphorylation process by still unknown Tyr-kinase(s) sensitive to kinase inhibitors such as ginestein or PP1, a specific SFK inhibitor, and phosphatase(s), most likely the Ser-Thr phosphatase family. In normal erythrocytes, the Na-K-2Cl cotransport related signaling pathway is also affected by oxygenation and deoxygenation conditions, further supporting the importance of the balance of kinase/phosphatase activities in red cell lifespan [19,118,122]. The K-Cl cotransport (KCC) is an electro-neutral gradient driven system modulated by cell age, cell swelling, pH, cell Mg content, and oxidation [19,118,122]. Increased K-Cl cotransport activity has been described in reticulocytes from healthy subjects as well as in reticulocytes and cells from patients with SCD or thalassemic syndromes. In these hemoglobinopathies the abnormal activation of the K-Cl cotransport has been related to the combination of severe membrane oxidative damage but also to the effect of relatively positively charged HbS on the transport itself or on one of its regulators [19,118,122]. Studies on red cell membrane permeability in SCD have shown that K-Cl cotransport plays a key role in generation of dense erythrocytes, particularly for sickle reticulocytes. Studies in both human and mouse models for SCD have further supported the importance of these dense, dehydrated red cells in the pathogenesis of both acute and chronic sickle cell related organ damage. Previous reports with inhibitors of family of phosphatases or kinases indicate that the activity of K-Cl cotransport is converted from its resting state to an activated state by a cascade of phosphorylation/dephosphorylation events [118,121,123]. Evidence from human sickle erythrocytes indicate the PP-1 family of phosphatases as activators of K-Cl cotransport [5,118,121,123]. Finally, we have shown that red cells from mice genetically lacking Src family tyrosine kinases Lyn, Hck, and Fgr display abnormally increased K-Cl cotransport activity and dehydrated erythrocytes [10]. Collectively, our data highlight the novel role of SFKs in down-regulating the K-Cl cotransport activity and inhibiting the K-Cl cotransport stimulator PP-1α. Interestingly, other studies have reported the role of erythroid Src and Syk kinases as modulators of K-Cl cotransport in human sickle erythrocytes exposed to cyclic oxy-deoxygenation [19,118,121]. Taken together, these data support the importance of signal transduction pathways in maintaining an optimal cell volume and red cell features during aging in the peripheral circulation. 

## 5. Conclusions

A fine balancing between kinases and phosphatases is crucial in cell homeostasis. Oxidative stress might affect cell signaling pathways directly by targeting either kinases or phosphatases, or indirectly by inducing the activation or overactivation of cytoprotective systems such as in CLL cells. SFKs are involved in different cellular processes such as maturation, growth, and survival. The over-activation or the intracellular accumulation of active SFKs, such as Lyn, results in severe perturbation of the dynamic coordination between kinases and phosphatases, deeply affecting cell homeostasis and cell survival. The intricate nature of intracellular signal transduction mechanisms, based on the generation of complex networks of different types of signaling proteins, revealed the novel and important role of phosphatases in disease mechanisms such as in CLL. Thus, therapeutic approaches to abnormal signal transduction pathways should consider either inhibition of overactivated/accumulated kinases or “homeostatic” signaling resetting. This is no longer based on single-target inhibitory drugs, such as Lyn-specific inhibitors, but on up-modulation of the physiological molecular counter balancer of protein kinases, that is protein phosphatases. Such “homeostatic” approaches to intracellular signal transduction modulation allow inducing an equilibrated “resetting” of imbalanced signaling mechanisms, thus eliminating the typical side effects of traditional pharmacological approaches based on mono-target therapies. In addition, potentiation of endogenous antioxidant system(s) might represent an attractive additional therapeutic strategy to limit prolonged/chronic oxidation involved in the pathogenesis of incurable disorders such as CLL or invalidating diseases such as β-thalassemia or SCD. Thus, future studies should be designed to better understand the dynamic coordination between kinases and phosphatases and its connection with oxidation in hematological disorders. This will allow progression beyond the state of the art and the possible identification of new drug targets.
